# The comparable tumour microenvironment in sporadic and *NF2*-related schwannomatosis vestibular schwannoma

**DOI:** 10.1093/braincomms/fcad197

**Published:** 2023-07-21

**Authors:** Grace E Gregory, Adam Paul Jones, Michael J Haley, Christopher Hoyle, Leo A H Zeef, I-Hsuan Lin, David J Coope, Andrew T King, D Gareth Evans, Pawel Paszek, Kevin N Couper, David Brough, Omar N Pathmanaban

**Affiliations:** Division of Neuroscience, School of Biological Sciences, Faculty of Biology, Medicine and Health, University of Manchester, Manchester Academic Health Science Centre, Manchester, UK; Geoffrey Jefferson Brain Research Centre, The Manchester Academic Health Science Centre, Northern Care Alliance NHS Foundation Trust, University of Manchester, Manchester, UK; The Lydia Becker Institute of Immunology and Inflammation, University of Manchester, Manchester, UK; Geoffrey Jefferson Brain Research Centre, The Manchester Academic Health Science Centre, Northern Care Alliance NHS Foundation Trust, University of Manchester, Manchester, UK; The Lydia Becker Institute of Immunology and Inflammation, University of Manchester, Manchester, UK; Division of Immunology, Immunity to Infection and Respiratory Medicine, Faculty of Biology, Medicine and Health, University of Manchester, Manchester, UK; Geoffrey Jefferson Brain Research Centre, The Manchester Academic Health Science Centre, Northern Care Alliance NHS Foundation Trust, University of Manchester, Manchester, UK; The Lydia Becker Institute of Immunology and Inflammation, University of Manchester, Manchester, UK; Division of Immunology, Immunity to Infection and Respiratory Medicine, Faculty of Biology, Medicine and Health, University of Manchester, Manchester, UK; Division of Neuroscience, School of Biological Sciences, Faculty of Biology, Medicine and Health, University of Manchester, Manchester Academic Health Science Centre, Manchester, UK; Geoffrey Jefferson Brain Research Centre, The Manchester Academic Health Science Centre, Northern Care Alliance NHS Foundation Trust, University of Manchester, Manchester, UK; The Lydia Becker Institute of Immunology and Inflammation, University of Manchester, Manchester, UK; Bioinformatics Core Facility, University of Manchester, Manchester, UK; Bioinformatics Core Facility, University of Manchester, Manchester, UK; Geoffrey Jefferson Brain Research Centre, The Manchester Academic Health Science Centre, Northern Care Alliance NHS Foundation Trust, University of Manchester, Manchester, UK; Department of Neurosurgery, Manchester Centre for Clinical Neurosciences, Salford Royal Hospital, Northern Care Alliance NHS Foundation Trust, Salford, UK; Geoffrey Jefferson Brain Research Centre, The Manchester Academic Health Science Centre, Northern Care Alliance NHS Foundation Trust, University of Manchester, Manchester, UK; Division of Cardiovascular Sciences, School of Biological Sciences, Faculty of Biology, Medicine and Health, University of Manchester, Manchester Academic Health Science Centre, Manchester, UK; Department of Neurosurgery, Manchester Centre for Clinical Neurosciences, Salford Royal Hospital, Northern Care Alliance NHS Foundation Trust, Salford, UK; Geoffrey Jefferson Brain Research Centre, The Manchester Academic Health Science Centre, Northern Care Alliance NHS Foundation Trust, University of Manchester, Manchester, UK; Division of Evolution and Genomic Sciences, School of Biological Sciences, Faculty of Biology, Medicine and Health, University of Manchester, Manchester Academic Health Science Centre, Manchester, UK; The Lydia Becker Institute of Immunology and Inflammation, University of Manchester, Manchester, UK; Division of Immunology, Immunity to Infection and Respiratory Medicine, Faculty of Biology, Medicine and Health, University of Manchester, Manchester, UK; Geoffrey Jefferson Brain Research Centre, The Manchester Academic Health Science Centre, Northern Care Alliance NHS Foundation Trust, University of Manchester, Manchester, UK; The Lydia Becker Institute of Immunology and Inflammation, University of Manchester, Manchester, UK; Division of Immunology, Immunity to Infection and Respiratory Medicine, Faculty of Biology, Medicine and Health, University of Manchester, Manchester, UK; Division of Neuroscience, School of Biological Sciences, Faculty of Biology, Medicine and Health, University of Manchester, Manchester Academic Health Science Centre, Manchester, UK; Geoffrey Jefferson Brain Research Centre, The Manchester Academic Health Science Centre, Northern Care Alliance NHS Foundation Trust, University of Manchester, Manchester, UK; The Lydia Becker Institute of Immunology and Inflammation, University of Manchester, Manchester, UK; Division of Neuroscience, School of Biological Sciences, Faculty of Biology, Medicine and Health, University of Manchester, Manchester Academic Health Science Centre, Manchester, UK; Geoffrey Jefferson Brain Research Centre, The Manchester Academic Health Science Centre, Northern Care Alliance NHS Foundation Trust, University of Manchester, Manchester, UK; The Lydia Becker Institute of Immunology and Inflammation, University of Manchester, Manchester, UK; Department of Neurosurgery, Manchester Centre for Clinical Neurosciences, Salford Royal Hospital, Northern Care Alliance NHS Foundation Trust, Salford, UK

**Keywords:** tumour microenvironment, vestibular schwannoma, tumour-associated macrophages, NF2, *NF2*-related schwannomatosis

## Abstract

Bilateral vestibular schwannoma is the hallmark of *NF2*-related schwannomatosis, a rare tumour predisposition syndrome associated with a lifetime of surgical interventions, radiotherapy and off-label use of the anti-angiogenic drug bevacizumab. Unilateral vestibular schwannoma develops sporadically in non-*NF2*-related schwannomatosis patients for which there are no drug treatment options available. Tumour-infiltrating immune cells such as macrophages and T-cells correlate with increased vestibular schwannoma growth, which is suggested to be similar in sporadic and *NF2*-related schwannomatosis tumours. However, differences between *NF2*-related schwannomatosis and the more common sporadic disease include *NF2*-related schwannomatosis patients presenting an increased number of tumours, multiple tumour types and younger age at diagnosis. A comparison of the tumour microenvironment in sporadic and *NF2*-related schwannomatosis tumours is therefore required to underpin the development of immunotherapeutic targets, identify the possibility of extrapolating *ex vivo* data from sporadic vestibular schwannoma to *NF2*-related schwannomatosis and help inform clinical trial design with the feasibility of co-recruiting sporadic and *NF2*-related schwannomatosis patients. This study drew together bulk transcriptomic data from three published Affymetrix microarray datasets to compare the gene expression profiles of sporadic and *NF2*-related schwannomatosis vestibular schwannoma and subsequently deconvolved to predict the abundances of distinct tumour immune microenvironment populations. Data were validated using quantitative PCR and Hyperion imaging mass cytometry. Comparative bioinformatic analyses revealed close similarities in *NF2*-related schwannomatosis and sporadic vestibular schwannoma tumours across the three datasets. Significant inflammatory markers and signalling pathways were closely matched in *NF2*-related schwannomatosis and sporadic vestibular schwannoma, relating to the proliferation of macrophages, angiogenesis and inflammation. Bulk transcriptomic and imaging mass cytometry data identified macrophages as the most abundant immune population in vestibular schwannoma, comprising one-third of the cell mass in both *NF2*-related schwannomatosis and sporadic tumours. Importantly, there were no robust significant differences in signalling pathways, gene expression, cell type abundance or imaging mass cytometry staining between *NF2*-related schwannomatosis and sporadic vestibular schwannoma. These data indicate strong similarities in the tumour immune microenvironment of *NF2*-related schwannomatosis and sporadic vestibular schwannoma.

## Teaser

Vestibular schwannoma are brain tumours found in *NF2*-related schwannomatosis patients and non-*NF2*-related schwannomatosis sporadic patients. Gregory *et al.* found strong similarities in the cell types, blood vessels, gene expression and inflammatory pathways of *NF2*-related schwannomatosis and sporadic vestibular schwannoma; thus, drugs and clinical trials may be equally effective in both groups.

## Graphical Abstract

**Graphical Abstract:**
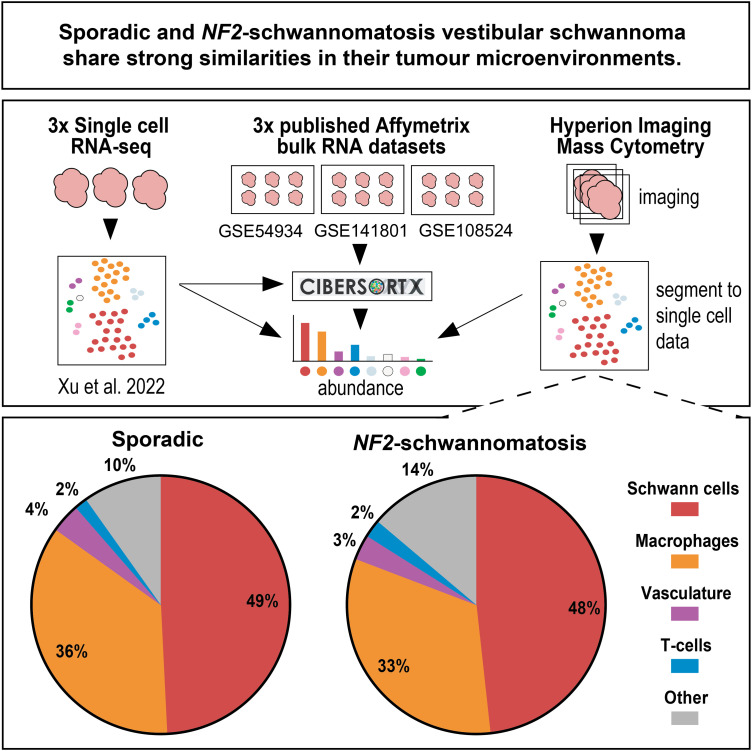

## Introduction

In the rare tumour predisposition syndrome *NF2*-related schwannomatosis (*NF2*-SWN)—previously known as neurofibromatosis type 2—pathogenic variants in the tumour suppressor *NF2* gene elicit the growth of bilateral vestibular schwannoma (VS). These neoplasms develop from Schwann cells that myelinate the eighth cranial nerve in the internal auditory meatus and cerebellopontine angle.^1^ In addition to *NF2*-SWN VS, mutations in *NF2* are also noted in 49–66% of the more common sporadic unilateral VS or up to 86% in 181 VS when loss of the *NF2* locus on 22q is included.^2–5^ Patients with VS, whether *NF2*-SWN or sporadic, present with sensorineural hearing loss, imbalance and tinnitus, which greatly reduce the quality of life. Surgical excision, radiotherapy and, for *NF2*-SWN patients only, treatment with the anti-angiogenesis drug bevacizumab are widely used,^6,7^ but all have limitations and risks; therefore, new options are needed. Given that *NF2*-SWN is a rare disease and sporadic tumours that need additional treatment options are also rare, it would be advantageous to find appropriate treatment targets applicable to both groups for preclinical and clinical trial drug development.

As well as genetic and symptom similarities between *NF2*-SWN VS and sporadic VS, their histopathology is synonymous with regions of cellular Antoni A, acellular Antoni B, scarring and whorls appearing equally in both, despite *NF2*-SWN VS tumours being multi-focal and more lobular in outward appearance than sporadic VS.^8^ Furthermore, the presence of a pathogenic variant in *NF2* in sporadic tumours appears not to affect bulk gene expression profiles of the affected sporadic VS compared with sporadic VS without alterations in *NF2.*^9^ Additionally, as the location of *NF2*-SWN and sporadic VS in the internal auditory meatus and cerebellopontine angle is identical, these tumours harbour the same potential interaction with the body’s immune system.^10^ However, there are potential differences including the presence of multiple schwannoma, meningioma and ependymoma in *NF2*-SWN versus solitary tumours in sporadic disease. Additionally, the immune system in *NF2*-SWN is likely to be exposed to schwannoma at earlier age.^11^ Finally, with the exception of some mosaic cases, all the immune cells of *NF2*-SWN patients will harbour a variant *NF2* allele.^12^ Inflammation is present in both sporadic and *NF2*-SWN VS with similar levels of tumour-associated leukocyte infiltration,^8,13,14^ and inflammation—specifically increased circulating chemokines and macrophage infiltration—correlates with increased sporadic VS growth.^15–18^ Along with macrophage niches, tumour-infiltrating lymphocytes such as T-cells and, to a much lesser extent, B cells are found within both sporadic and *NF2*-SWN VS.^19,20^ Both macrophages and T-cells within the tumour immune microenvironment have garnered attention for their potentials as therapeutic targets in VS,^10,21,22^ but as yet, no studies have aimed to identify whether targeted immunotherapeutics would be equally efficacious in both sporadic and *NF2*-SWN VS, nor whether the more common sporadic VS samples could be used to assess therapeutics to treat *NF2*-SWN VS and vice versa.

The study presented here aimed to interrogate and compare the immune compartments between *NF2*-SWN and sporadic VS to characterize similarities or differences in their tumour immune microenvironments. This study drew together bulk transcriptomic data from three published Affymetrix microarray datasets (GSE54934, GSE108524 and GSE141801) to assess the broad gene expression profiles and functional signalling pathways of *NF2*-SWN VS compared to sporadic VS. Additionally, the bulk transcriptomic data were deconvolved using a novel VS-specific signature matrix generated and validated in this study for CIBERSORTx.^23,24^ This was used to predict cell type abundances within sporadic and *NF2*-SWN VS tumour microenvironments. Marker expression was validated using quantitative PCR (qPCR). Additionally, this study was the first to implement high-dimensional imaging mass cytometry (IMC) to investigate the tumour microenvironment which reinforced the prominence of Schwann cells and macrophages in both sporadic and *NF2*-SWN, with comparable abundances observed in both tumour types.

Together, these IMC data with the bulk transcriptomic expression profiles, cell abundance deconvolution and qPCR highlight the strong similarities between the cell type abundances of *NF2*-SWN and sporadic VS. Therefore, targeted immunotherapies may be equally effective in both sporadic and *NF2*-SWN VS and it would be reasonable to recruit both patent cohorts to future immune-targeted clinical trials.

## Materials and methods

### Affymetrix microarray data extraction and differential gene expression analyses

Publicly available Affymetrix microarray datasets GSE54934,^9^ GSE108524^25^ and GSE141801^26^ were downloaded from Gene Expression Omnibus. Clinical information on patient samples in these datasets can be found in the Gene Expression Omnibus repositories and associated published studies for GSE54934,^9^ GSE108524^25^ and GSE141801.^26^ Samples with confirmed irradiation prior to resection were excluded for this study. Microarray data from all three datasets were pre-processed by performing Robust Multichip/multi-array Analysis (RMA) using the R Bioconductor package, ‘affycoretools’.^27^ Annotation of GSE54934, GSE108524 and GSE141801 was completed with the U219 array database ‘pd.hugene.1.0.st.v1’,^28^ the Human Transcriptome Array database ‘pd.hta.2.0’,^29^ and the U219 array database ‘pd.hg.u219’,^30^ respectively. All differential gene expression analysis was conducted using R package ‘limma’ with empirical Bayes.^31^ For GSE54934, differential gene expression analysis was conducted on three groups (control vestibular and eighth cranial nerve *n* = 2, sporadic VS *n* = 28 and *NF2*-SWN VS *n* = 3). For GSE108524, differential gene expression analysis was conducted on three groups (control vestibular nerve *n* = 4, sporadic VS *n* = 10 and *NF2*-SWN VS *n* = 17). For GSE141801, differential gene expression analysis was conducted on three groups (control vestibular nerve *n* = 7, sporadic VS *n* = 45 and *NF2*-SWN VS *n* = 13). Subsequently, principal component analysis (PCA) was performed on sporadic and *NF2*-SWN samples. Expression was averaged per gene to reduce probe set variation in cases where multiple probes were present for each gene. Hierarchical clustering of relative mean gene expression for heatmap generation used one minus Pearson’s correlation.

### Functional enrichment analysis

Lists of differentially expressed genes (DEG), their FDR-adjusted *P*-values and absolute fold changes enriched between nerve and sporadic VS or nerve and *NF2*-SWN VS within each of the three datasets were uploaded into Ingenuity Pathway Analysis (IPA) (Qiagen). IPA’s ‘Core analysis’ function was used to retrieve significantly dysregulated canonical pathways according to the Ingenuity Pathway Knowledge Base.

### Bulk gene expression deconvolution

To determine immune cell composition in VS, single-cell RNA sequencing (scRNA-seq) data from three sporadic VS samples from Xu *et al*. (2022) were obtained from the National Omics Data Encyclopaedia (https://www.biosino.org/node; accession code OEP001871). Clinical information on patient samples can be found in the associated published Xu *et al*. (2022) study.^32^ Data were processed using various R packages (v4.1).^32^ Briefly, Scuttle R package (v1.4.0) was used to compute per-cell and per-gene quality control metrics in each sample from the expression matrices. Median absolute deviation was implemented in the Scuttle R package for exact thresholds to identify and subsequently remove poor-quality cells before further processing. The log-normalized expression values of each sample were re-calculated using the batchelor R package (v1.10.0) to adjust for the systematic differences in coverage between them. Graph-based clustering of cells using the Louvain algorithm from the igraph R package (v1.3.0) with the bluster R package (v1.4.0) identified 18 non-doublet clusters. Known marker genes of various cell types such as Schwann cells, macrophages, fibroblasts, endothelial cells and other immune cells were used to manually annotate the clusters into distinct cell types (Supplementary Table 1).

The human gene annotation was obtained from Ensembl (Homo_sapiens.GRCh38.98.gtf) to calculate the exonic gene lengths to normalize scRNA-seq raw counts into transcripts per million (TPM) values. From the TPM scRNA-seq data, the ‘random’ python package randomly sampled 25% of the single cells that were used to generate a VS-specific signature matrix and gene expression profile (GEP) file with CIBERSORTx (https://cibersortx.stanford.edu/).^23,24^ The remaining 75% of the single cells were used to generate pseudo-bulk data by averaging known proportions of single-cell TPM of the annotated clusters, e.g. 25:75 Schwann cells:macrophages. These pseudo-bulk tissues were used to validate the estimated proportions of cell types from the pseudo-bulk sample using the VS-specific signature matrix in the deconvolution algorithm CIBERSORTx as seen in Supplementary Fig. 1.^33,34^ The VS-specific signature matrix and GEP files generated in this study were made publicly available using FigShare.^24^ To run CIBERSORTx, batch correction was enabled in ‘bulk mode’ in absolute at 1000 permutations.

The annotated Affymetrix RMA gene expression data for datasets GSE54934, GSE108524 and GSE141801 in TPM were used to predict the estimated proportions of cell types from the bulk samples CIBERSORTx.^33,34^ The VS-specific signature matrix and GEP files were used with batch correction enabled in ‘bulk mode’ and then run in absolute at 1000 permutations in CIBERSORTx.^24^

### qPCR

Fresh tumour samples from 15 sporadic VS (7 male, 8 female) and 4 *NF2*-SWN VS (4 female) were collected under the Northern Care Alliance Research Collection (NCARC, REC reference 18/WA/0368) in Dulbecco’s modified Eagle medium (Thermo Fisher Scientific). Samples were washed in phosphate-buffered saline (Sigma-Aldrich) before being stored in RNA Protect (Qiagen) at −80°C. Tumour samples were homogenized, and RNA was extracted using the RNeasy Lipid Tissue Mini Kit (Qiagen). After RNA extraction, cDNA was synthesized using Superscript III Reverse Transcriptase (Thermo Fisher) according to the manufacturer’s instructions. Primer pairs were designed using Primer-BLAST and ordered from Invitrogen. Primer sequences used were as follows: *TNF*: forward 5′-TGCACTTTGGAGTGATCGGC-3′, reverse 5′-AGCTTGAGGGTTTGCTACAAC-3′; *IL6*: forward 5′-CATCCTCGACGGCATCTCAG-3′, reverse 5′-CACCAGGCAAGTCTCCTCATT-3′; *IL18*: forward 5′-AAGGAAATGAATCCTCCTGATAACA-3′, reverse 5′-CCTGGGACACTTCTCTGAAAGAA-3′; *NLRP3*: forward 5′-TGCCCGTCTGGGTGAGA-3′, reverse 5′-CCGGTGCTCCTTGATGAGA-3′; *IL1B*: forward 5′-ACGATGCACCTGTACGATCACT-3′, reverse 5′-CACCAAGCTTTTTTGCTGTGAGT-3′; *CASP1*: forward 5′-ATACCAAGAACTGCCCAAGTTTG-3′, reverse 5′-GGCAGGCCTGGATGATGA-3′; *CD163*: forward 5′-AAAAAGCCACAACAGGTCGC-3′, reverse 5′-CTCTTGAGGAAACTGCAAGCC-3′; *MRC1*: forward 5′-TGCTACTGAACCCCCACAAC-3′, reverse 5′-ACCAGAGAGGAACCCATTCG-3′; *CD68*: forward 5′-CCTCAGCTTTGGATTCATGCAG-3′, reverse 5′-GAGAATGTCCACTGTGCTGCG-3′; *GNB2L1*: forward 5′-CTTCTGGAGGCAAGGATGGC-3′, reverse 5′-CACACAGCCAGTAGCGGTTA-3′. Samples were run in triplicate using Power SYBR Green PCR master mix (Thermo Fisher) and 200 nM of each primer using a 7900HT Fast Real-Time PCR System (Applied Biosystems). For each primer set, the efficiency was >90% and a single product was seen during melt curve analysis. Relative expression levels were calculated using the 2^−ΔΔCt^ method normalizing to the expression of the housekeeping gene *GNB2L1*.

### Tissue staining for imaging mass cytometry

Nine sporadic VS samples (7 male, 2 female) and 13 *NF2*-SWN VS samples (6 male, 7 female) were accessed through the NCARC (REC reference 18/WA/0368; IRAS ID 145069) and Health Research Authority and Health and Care Research Wales (REC reference 20/NW/0015; IRA ID 274046). All sporadic and *NF2*-SWN VS tumours used for imaging mass cytometry were naïve with no prior radiation or bevacizumab treatment. Samples were stored as formalin-fixed paraffin embedded blocks from which 5-*µ*m VS tissue was cut and fixed onto individual standard slides for sporadic samples, or 2-mm diameter cores from *NF2*-SWN to create a tissue microarray as determined by histopathological features present (Antoni A, Antoni B, abnormal vasculature) by a neuropathologist. For deparaffinization, slides were submerged in xylene for 10 min followed by 1-min submersion in xylene then ethanol at 100, 90, 70 and 50%, before being placed in ultrapure water for 5 min. After rehydration, slides were incubated at 96°C for 30 min in excess Tris-EDTA at pH 8.5. Slides were cooled to 70°C and washed with phosphate-buffered saline (PBS) for 8 min. The tissues on the slides were outlined by a hydrophobic barrier pen, and then, 3% bovine serum albumin (BSA) in PBS was added on the tissue for 45 min. Biotin block was applied for 15 min followed by a streptavidin block for 15 min. All lanthanide-conjugated antibodies were diluted in PBS with 0.5% bovine serum albumin as a master mix as shown in Supplementary Table 2. Antibodies had been validated with positive control tissue (secondary lymphoid organs for immune-targeted antibodies and lung tumour samples for neoplastic markers) and dilutions optimized with VS tissue. The antibody master mix was applied on the slides and incubated overnight at 4°C in a hydration chamber. After overnight incubation, slides were washed with PBS for 8 min, repeated twice. Slides were then washed with 0.1% Triton-X100 in PBS for 8 min, repeated twice followed by one wash with PBS for 5 min. Iridium was diluted at 1:400 in PBS and added to the slides for 30 min at RT. After iridium staining, slides were washed with ultrapure water for 10 seconds and air dried for 20 min.

### Imaging mass cytometry

Regions of interest (sporadic VS: 6 per case, each 1060 × 1060 *µ*m, *NF2*-SWN VS: 3–8 per case, each 1000 × 1000 *µ*m) for IMC imaging were identified on H&E-stained sections from each case and were selected by a neuropathologist. IMC images of tissue sections bound with metal-conjugated antibodies were acquired using a Hyperion imaging mass cytometer (Standard Biotools). In brief, the tissue was laser ablated in a rastered pattern in a series of 1-*µ*m^2^ pixels. The resulting plume of ablated tissue was then passed through a plasma source, ionizing it completely into its constituent atoms. Time-of-flight mass spectrometry then discriminated the signal for each of the metal-conjugated antibodies, and images for each antibody were reconstructed based off the metal abundancy at each pixel. Staining was reviewed using MCD Viewer (Standard Biotools), with regions of interest with unsuccessful staining excluded from further analysis. Images (.tiff) images were exported from MCD Viewer, and Fiji was used to visualize the staining of S100B, Iba1, smooth muscle actin (SMA), CD8a and von Willebrand Factor (vWF).

### Cell segmentation

Single-cell information was extracted from the images produced by the Hyperion imaging mass cytometry system using a published protocol.^35^ In brief, stacks of TIFF images were constructed for each region of interest whereby individual channels corresponded to each lanthanide-conjugated antibody. Ilastik was then used to produce a pixel probability classifier that identified background, cytoplasmic and nuclear pixels.^36^ The resulting pixel probability maps were then converted into cell segmentation masks that identified the regions corresponding to individual cell boundaries. These cell segmentation masks were then applied to each of the antibody channels, generating single-cell expression data for each of the channels, along with the spatial context of where the cell was located in the region of interest.

### Single-cell data quantification

Single-cell data was interrogated using a bespoke Python pipeline for analysing IMC data that uses the Scanpy ecosystem.^37^ Initially, single-cell mean intensities were normalized to the 99th percentile, thereby scaling marker intensities between 0 and 1. Leiden clustering was then used to identify cell populations present in the IMC data, which were then manually annotated based upon known patterns of marker expression corresponding to known cell types—Schwann cells, macrophages, vascular cells or T-cells, with unidentifiable populations labelled as ‘other’. In some cases, Leiden clusters were merged if they identified the same underlying biological cell populations. The resulting biological populations and expression of individual markers were then visualized on a UMAP projection of the data. Populations were then spatially mapped to their location in the tissue for each region of interest.

### Statistical analysis

When assessing *NF2*-SWN VS RNA expression relative to sporadic VS in the three publicly available Affymetrix datasets, *P*-values were adjusted for multiple comparisons using the Benjamini–Hochberg method to control for false discovery rate (FDR).^38^ Genes were considered to be DEGs if the FDR-adjusted *P*-value was ≤0.01 with a fold change of ≥2 or ≤−2. The normality of distributions of relative RNA expression in GSE108524, GSE54934 and GSE141801 was determined by the Shapiro–Wilk test. Subsequently, a parametric two-way analysis of variance (ANOVA) with Bonferroni adjustment for multiple comparisons was used and statistical significance was considered when *P* ≤ 0.05.

For functional enrichment analysis, significantly dysregulated canonical pathways were determined when compared to the Ingenuity Pathway Knowledge Base by using a right-tailed Fisher’s exact test for *P*-value generation and *z*-score for the predicted activity or inactivity of the pathways.

For the predicted cell type proportions derived from CIBERSORTx, the normality of distribution of the data was determined by the Shapiro–Wilk test. Subsequently, the Mann–Whitney *U* test with the Benjamini–Hochberg method to control for FDR was applied to all three datasets.^38^ Statistical significance was considered when FDR-corrected *P* ≤ 0.05.

In qPCR analysis, normality of the distribution of RNA expression relative to sporadic VS was determined by the Shapiro–Wilk test followed by a parametric two-way ANOVA with Bonferroni adjustment. Statistical significance was considered at *P* ≤ 0.05.

For Hyperion IMC, the normality of distribution of cell abundance data for *NF2*-SWN and sporadic samples was determined by the Shapiro–Wilk test. Subsequently, a parametric two-way ANOVA with Bonferroni adjustment for multiple comparisons was used with statistical significance at *P* ≤ 0.05.

## Results

### Vestibular schwannoma is enriched for immune gene expression compared to vestibular nerve

**Figure 1:**
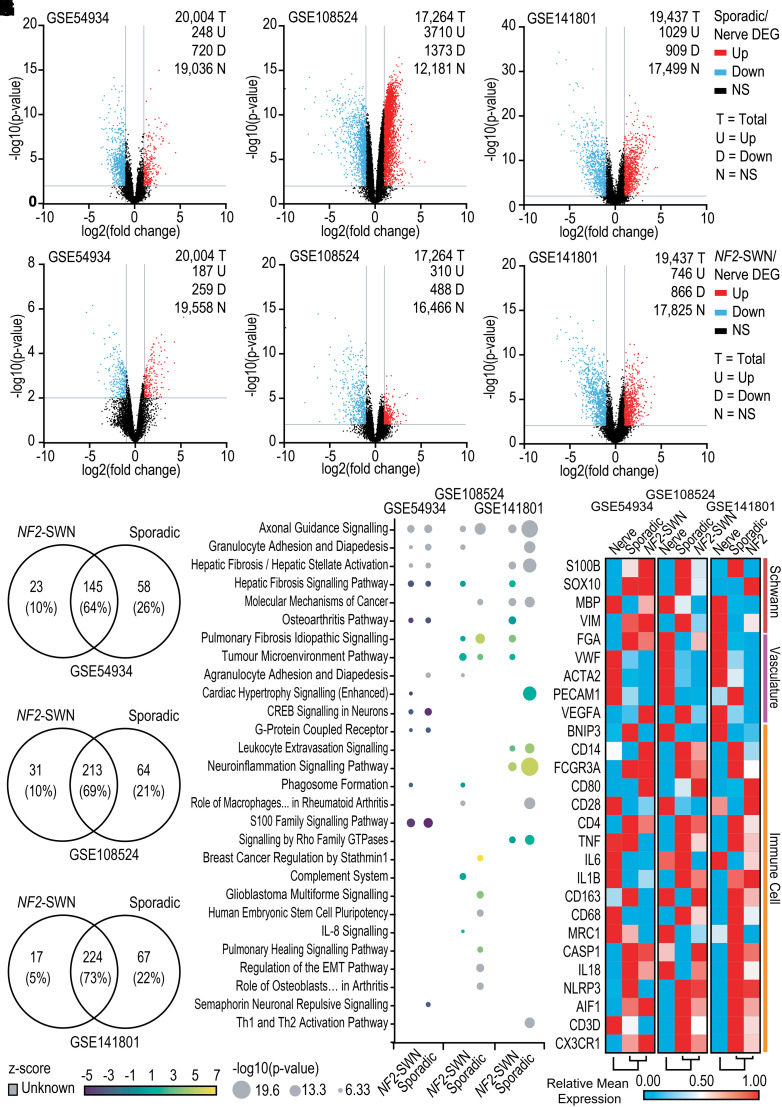
**Sporadic and *NF2*-schwannomatosis (*NF2*-SWN) vestibular schwannoma (VS) are enriched in immune-related pathways and immune gene expression compared to healthy vestibular nerve.** All data acquired from GSE54934, GSE108524 and GSE141801 are available from Gene Expression Omnibus. GSE54934 included control vestibular and eighth cranial nerve *n* = 2, sporadic VS *n* = 28 and *NF2*-SWN VS *n* = 3. GSE108524 included control vestibular nerve *n* = 4, sporadic VS *n* = 10 and *NF2*-SWN VS *n* = 17. GSE141801 included control vestibular nerve *n* = 7, sporadic VS *n* = 45 and *NF2*-SWN VS *n* = 13. (**A–F**) Volcano plots identified numerous differentially expressed genes (DEGs) either up or downregulated in sporadic VS (**A–C**) or *NF2*-SWN VS (**D–F**) compared to nerve samples. *P* ≤ 0.01 with fold change of ≤−2 or ≥2. (**G–I**) The number of overlapping signalling pathways identified in *NF2*-SWN and sporadic VS compared to nerve using Ingenuity Pathway Analysis (IPA) ‘Core Analysis’. *P* ≤ 0.05. (**J**) Overlap between *NF2*-SWN and sporadic VS compared to nerve within and between datasets when assessing the top 10 pathways by significance (lowest *P*-values). *z*-score for predicted activity of the pathway. (**K**) Gene expression associated with Schwann cell, vasculature and immune cell profiles determined using IPA and literature search was assessed in *NF2*-SWN VS, sporadic VS and healthy nerve. One-minus Pearson correlation for hierarchical clustering. Genes named by Human Gene Nomenclature. Not significant (NS); cAMP response element-binding protein (CREB); epithelial-mesenchymal transition (EMT).

In order to compare gene expression profiles of *NF2*-SWN and sporadic VS compared to control vestibular nerve samples, DEG analysis was performed on three publicly available datasets: GSE54934, GSE108524 and GSE141801. Derived from the Schwann cells myelinating the eighth cranial nerve, VS have the same anatomical location and potential for immune interaction as the control nerve. However, as determined from datasets GSE54934, GSE108524 and GSE141801, both *NF2*-SWN and sporadic VS have different gene expression profiles compared to vestibular nerve. For example, when comparing the gene expression profiles of sporadic VS against nerve in GSE54934, GSE108524 and GSE141801, there were 968 (4.9%), 5083 (29%) and 1938 (10%) DEGs in sporadic VS compared to nerve, respectively (Fig. 1A–C). For *NF2*-SWN VS compared to vestibular nerve, a lower but still notable number of DEGs were observed with 446 (2.2%), 798 (4.6%) and 1612 (8.3%) DEGs in datasets GSE54934, GSE108524 and GSE141801, respectively (Fig. 1D–F).

Despite the lower number of DEGs in *NF2*-SWN, functional enrichment analysis in IPA revealed a robust overlap in significantly enriched functional pathways between *NF2*-SWN and sporadic VS when compared against nerve (Fig. 1G–I). The top 10 enriched canonical signalling pathways in *NF2*-SWN and sporadic VS, as indicated by their *P*-values, display strong similarity of the enriched pathways in the VS samples (Fig. 1J). Across the three datasets, there was a strong representation of genes in pathways related to immune cell function, extravasation and adhesion (Fig. 1J). This was highlighted by the neuroinflammation signalling pathway and tumour microenvironment pathway in both *NF2*-SWN and sporadic VS displaying high predicted activation of genes related to proliferation of tumour-associated macrophages, angiogenesis, inflammation and T-cell immunity when compared to nerve in GSE141801 and GSE108524. Interestingly, in all three datasets, fibrosis-related pathways and the axonal guidance signalling pathway were enriched in both *NF2*-SWN and sporadic VS when compared against nerve. This was likely due to an increased expression of proteins involved with cell adhesion observed in the pathway in IPA, such as the semaphorins, integrins and cell adhesion molecules linked to immune cell recruitment and extravasation, rather than in axonal guidance.

Additionally, to explore the gene expression of tumour microenvironment signatures, a panel of genes determined from the literature and IPA’s ‘Molecules’ function to represent Schwann cells,^39,40^ vasculature^41,42^ and immune cells^43–47^ were assessed in nerve, *NF2*-SWN VS and sporadic VS. Across all three datasets, gene expression associated with immune cell lineage was more highly expressed in sporadic and *NF2*-SWN VS compared to nerve (Fig. 1K). Overall, when comparing *NF2*-SWN and sporadic VS against nerve samples, the similarities in immune-related signalling pathways and gene expression imply a rich immune component to the tumour microenvironment in both *NF2*-SWN and sporadic VS.

### Sporadic and *NF2*-SWN VS have highly conserved gene expression profiles

**Figure 2:**
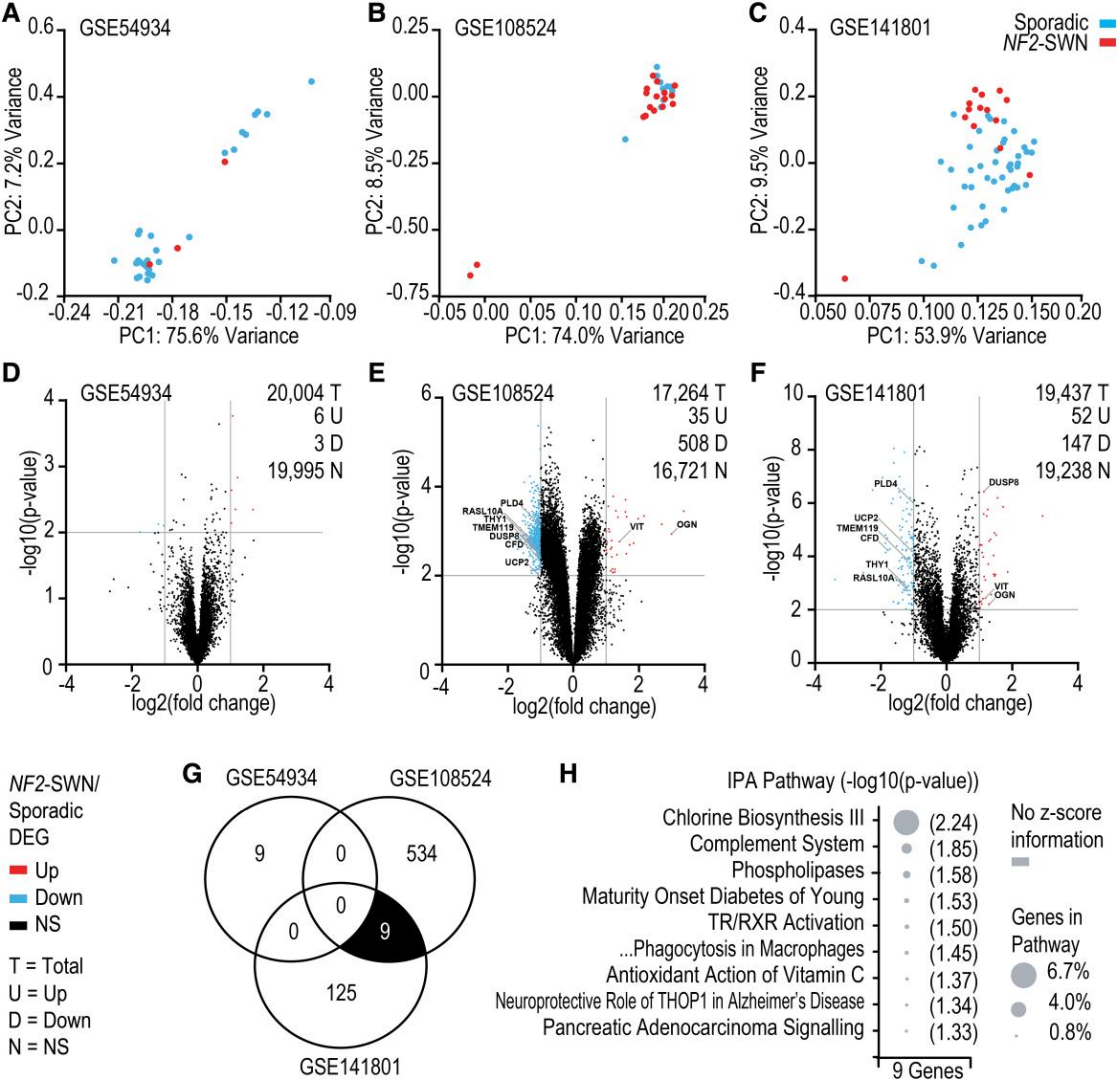
**NF2-schwannomatosis (*NF2*-SWN) compared with sporadic vestibular schwannoma (VS) display similarity in gene expression and signalling pathways.** GSE54934, GSE108524 and GSE141801 datasets were available from Gene Expression Omnibus. GSE54934 included control vestibular and eighth cranial nerve *n* = 2, sporadic VS *n* = 28 and *NF2*-SWN VS *n* = 3. GSE108524 included control vestibular nerve *n* = 4, sporadic VS *n* = 10 and *NF2*-SWN VS *n* = 17. GSE141801 included control vestibular nerve *n* = 7, sporadic VS *n* = 45 and *NF2*-SWN VS *n* = 13. (A–C) Principal component analyses (PCA) for dimensionality reduction display a strong overlap of *NF2*-SWN and sporadic VS samples by principal component 1 (PC1). (D–F) Volcano plots visualising the differentially expressed genes (DEG) between *NF2*-SWN and sporadic VS. (G) Overlap of DEGs between *NF2*-SWN and sporadic VS across the three datasets. The nine overlapping DEGs between GSE108524 and GSE141801 are annotated on the volcano plots (E and F). (H) Ingenuity Pathway Analysis ‘Core Analysis’ of the nine overlapping genes in (G). *P*-value ≤0.05 and z-score for predicted activity of the pathway. Abbreviations: thyroid hormone receptor/retinoid X receptor (TR/RXR), thimet oligopeptidase 1 (THOP1).

After comparing *NF2*-SWN and sporadic VS against nerve, a direct comparison of the *NF2*-SWN and sporadic VS was undertaken. *NF2*-SWN and sporadic VS samples overlapped strongly according to principal component 1 in principal component analysis (Fig. 2A–C). The divide in cases and experimental outliers seen in Fig. 1A–C likely relates to experimental variability but was not due to batch effects for analysis. Furthermore, very few significant DEGs existed between *NF2*-SWN compared to sporadic VS (Fig. 2D–F). Firstly, out of the 20 004 genes in GSE54934, 9 (0.055%) genes had significantly different expression compared to sporadic VS (Fig. 2D). Secondly in GSE108524, from the total 17 264 genes assessed, 543 (3.1%) genes were significant DEGs in *NF2*-SWN VS (Fig. 2E). Finally, of the 19 437 genes investigated in GSE141801, only 199 (1.0%) genes were significant DEGs in *NF2*-SWN VS compared to sporadic VS (Fig. 2F).

Importantly, there were no conserved DEGs when comparing *NF2*-SWN and sporadic VS across the three datasets, indicating the gene expression profile proposed to distinguish these VS was inconsistent. However, two datasets GSE108524 and GSE141801 did display nine shared DEGs (Fig. 2G, annotated in Fig. 2E and F, respectively). Interestingly, one gene—dual-specificity phosphatase 8 (*DUSP8*)*—*was not significant in GSE54934 but was significantly downregulated in GSE108524 and significantly upregulated in GSE141801 (Fig. 2E and F), reinforcing the gene expression profile inconsistency between the three datasets. As no significant IPA pathway contained more than one of the nine genes, they only accounted for a very small percentage of the total genes within the pathways, and therefore, the nine genes were unlikely to represent a biologically relevant difference between *NF2*-SWN and sporadic VS (Fig. 2H).

These data displayed in Fig. 2D–H signify that when each dataset is taken individually, DEG profiles can be ascertained for *NF2*-SWN and sporadic VS; however, by comparing these expression profiles, there are numerous differences in DEGs between datasets which display inconsistency. This lack of congruency in the DEG profile between datasets GSE54934, GSE108524 and GSE141801 was continued in functional enrichment analyses using IPA (Supplementary Fig. 2A–C), where there was little overlap in the predicted canonical pathways significantly different between *NF2*-SWN and sporadic VS (Supplementary Fig. 2D).

Together, these bulk transcriptomic data display very few common DEGs and pathways between the three data sets, suggesting that there is no distinct biological difference between *NF2*-SWN and sporadic VS. Thus, these data infer a broad similarity in gene expression and signalling pathways between *NF2*-SWN and sporadic VS.

### Sporadic and *NF2*-SWN VS comprise similar tumour microenvironment components

**Figure 3:**
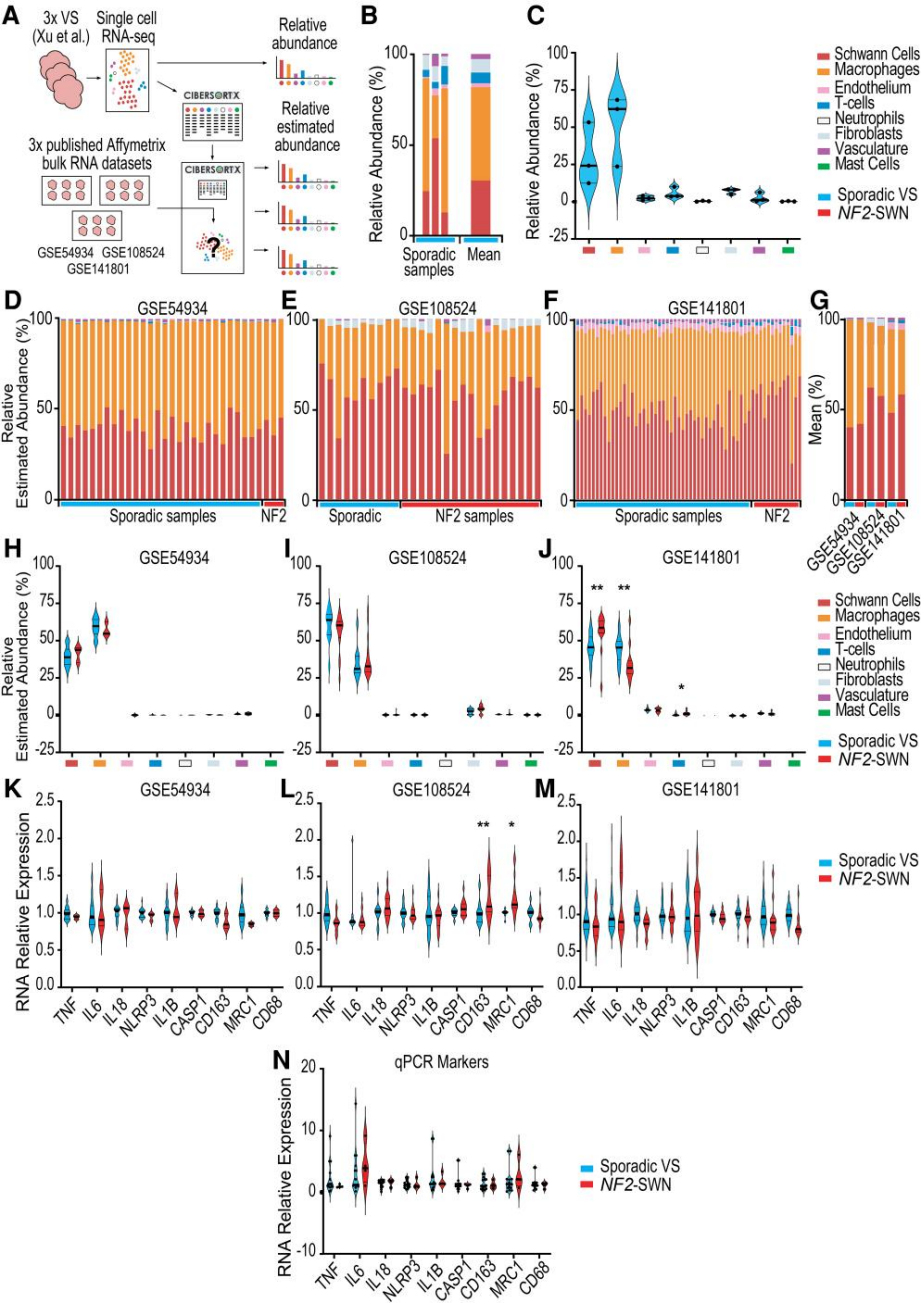
**Schwann cells and macrophages are the most abundant cell types in sporadic and *NF2*-related schwannomatosis (*NF2*-SWN) vestibular schwannoma (VS).** (**A, C**) Workflow for the use of single-cell RNA data (sporadic VS *n* = 3) published by Xu *et al.* (2022)^32^ available at National Omics Data Encyclopaedia accession code OEP001871 used (**A**) to identify relative cell abundance (**B, C**) and create new CIBERSORTx signature matrix.^24^ All data acquired from GSE54934, GSE108524 and GSE141801 available from Gene Expression Omnibus. GSE54934 included control vestibular and eighth cranial nerve *n* = 2, sporadic VS *n* = 28 and *NF2*-SWN VS *n* = 3. GSE108524 included control vestibular nerve *n* = 4, sporadic VS *n* = 10 and *NF2*-SWN VS *n* = 17. GSE141801 included control vestibular nerve *n* = 7, sporadic VS *n* = 45 and *NF2*-SWN VS *n* = 13. (**D–G**) Relative estimated abundance of cell populations by bulk RNA expression deconvolution (**D–F**) and mean estimated abundance across all three datasets (**G**). (**H–J**) Relative estimated abundance of each cell population in each of the three datasets. (**K–M**) The expression of macrophage marker genes between *NF2*-SWN and sporadic VS in each of the three datasets. (**N**) Quantitative PCR (qPCR) analysis of macrophage marker gene expression between *NF2*-SWN and sporadic VS in an additional cohort of VS (*n* = 4 *NF2*-SWN and *n* = 15 sporadic VS). Relative RNA expression was calculated using the 2^−ΔΔCt^ method normalizing to the expression of the housekeeping gene *GNB2L1*. Normality of the distributions of abundance and RNA expression relative to sporadic VS were determined by the Shapiro–Wilk test followed by multiple Mann–Whitney *U* test with Benjamini–Hochberg adjustment or a parametric two-way ANOVA with Bonferroni adjustment, respectively. Statistical significance *P* *≤* 0.05 (*) and *P* *≤* 0.01 (**).

Single-cell data from three sporadic VS published by Xu *et al.* (2022)^32^ were used to identify immune abundance as well as create a novel CIBERSORTx signature matrix for deconvolving bulk RNA expression data (Fig. 3A).^24^ These single-cell data display heterogeneity between samples, which on average were primarily composed of 30% Schwann cells and 51% macrophages (Fig. 3B and C). Using the novel signature matrix derived from the single-cell data, bulk expression data from the three Affymetrix microarray datasets GSE54934, GSE108524 and GSE141801 were deconvolved by CIBERSORTx to investigate the cell type composition within the tumour microenvironment. Across the three datasets and in every sample assessed, Schwann cells and macrophages were predicted to be of highest abundance (Fig. 3D–F), averaging between 40–61% and 36–59% in sporadic VS and 42–58% and 35–57% in *NF2*-SWN VS, respectively (Fig. 3G). There were no statistically significant differences in the relative estimated abundances of immune cell types between *NF2*-SWN and sporadic VS for any of the eight cell types detected in datasets GSE54934 and GSE108524 (Fig. 3H and I). However, in dataset GSE141801, macrophages were less abundant in *NF2*-SWN compared to sporadic VS but *NF2*-SWN VS were found to have a significantly greater proportion of T-cells (Fig. 3J). Again, these data highlight differences between the published datasets GSE54934, GSE108524 and GSE141801 but suggest no clear difference between *NF2*-SWN and sporadic VS, with similar estimated abundances of cell types within the tumour predominantly composed of Schwann cells and macrophages.

As macrophages were predicted to be the most abundant immune cell type in both *NF2*-SWN and sporadic VS in datasets GSE54934, GSE108524 and GSE141801, the RNA expression of markers associated with leukocyte function and polarization towards pro- or anti-inflammatory macrophage states were investigated (Fig. 3K–M). There was no significant difference in expression of the general myeloid marker gene *CD68* in the three datasets between *NF2*-SWN and sporadic VS. Genes associated with a pro-inflammatory tumour microenvironment included *TNF*, *IL6*, *IL18*, *NLRP3*, *IL1B* and *CASP1* which were also comparable between sporadic and *NF2*-SWN VS samples. However, solely in dataset GSE108524, there was a significant difference in anti-inflammatory macrophage marker genes *CD163* and *MRC1*, which exhibited increased expression in *NF2*-SWN VS over their sporadic counterparts (Fig. 3L). This difference was not found in datasets GSE54934 and GSE141801 (Fig. 3K and M). These findings were directly assessed and validated in a new sample cohort using qPCR, which also identified no significant differences between *NF2*-SWN and sporadic VS (Fig. 3N). Taken together, the bulk transcriptomic expression and deconvolved cell abundance data followed by qPCR indicate a broad similarity between the composition of the immune cell compartments and macrophage polarization in both *NF2*-SWN and sporadic VS.

### Hyperion IMC confirms the tumour microenvironment predominantly comprises Schwann cells and macrophages

**Figure 4:**
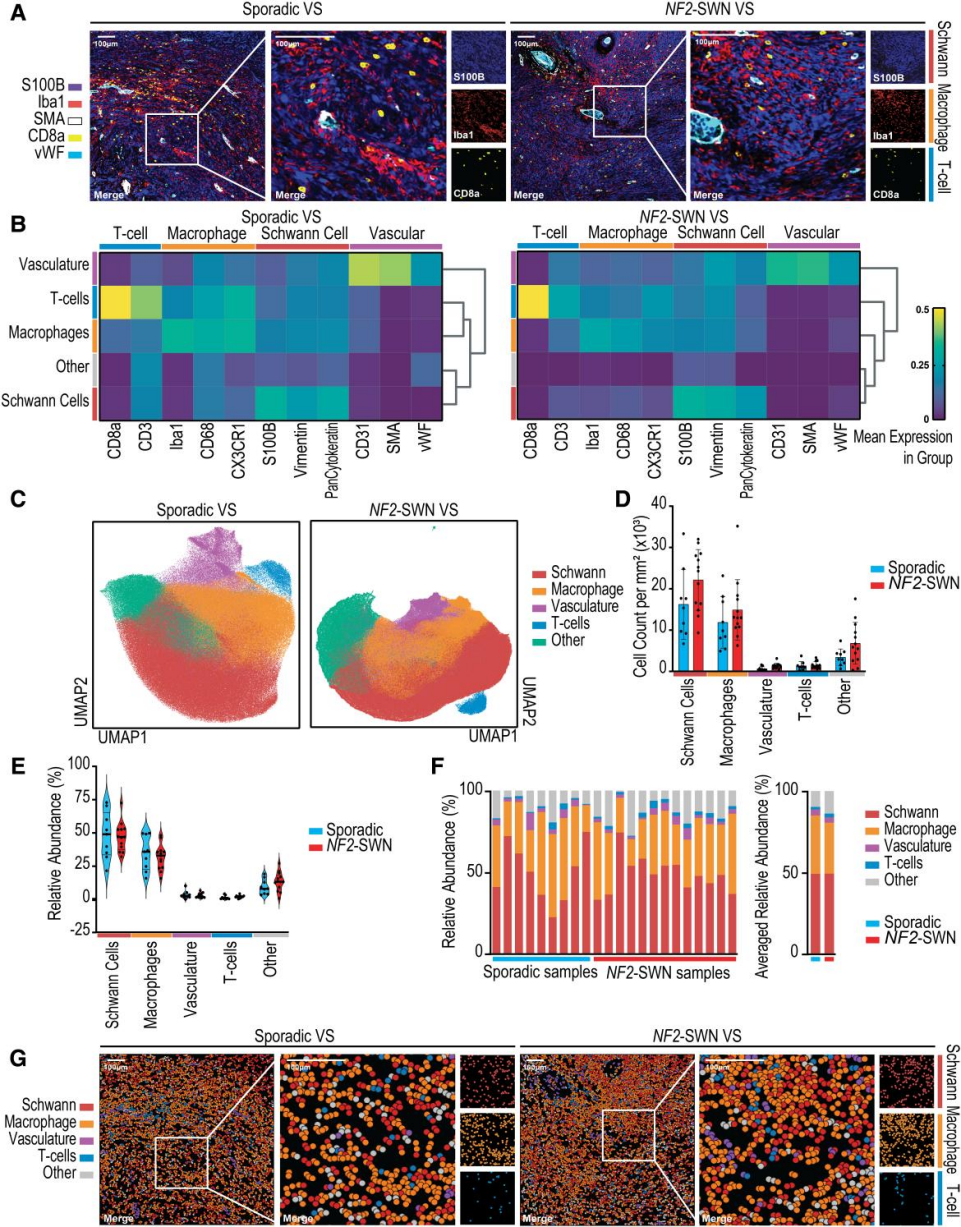
**Macrophages are the most abundant immune cell type, forming niches with infiltrating T-cells in sporadic and *NF2*-schwannomatosis (*NF2*-SWN) vestibular schwannoma (VS).** (**A**) Representative IMC images of S100B, Iba1, smooth muscle actin (SMA), CD8a and von Willebrand Factor (vWF) within sporadic and *NF2*-SWN VS. (**B**) Biological populations from *n* = 9 sporadic VS and *n* = 13 *NF2*-SWN VS in IMC data identified using Leiden clustering (**C**) UMAP projections of the single-cell IMC data coloured with the biological populations (**D**). Absolute cell type abundances between sporadic and *NF2*-SWN VS tumours. (**E, F**) Relative abundance of cell types within each VS averaged for sporadic and *NF2*-SWN. (**G**) Spatial mapping of single cells annotated by population visualized in *NF2*-SWN and sporadic VS. Normality of distribution of cell type abundance was determined by the Shapiro–Wilk test. Subsequently, a parametric two-way ANOVA with Bonferroni adjustment for multiple comparisons was used with statistical significance at *P* *≤* 0.05.

After the prediction of a high level of macrophage infiltration in sporadic and *NF2*-SWN VS tumours from bulk transcriptomic data, the cell type abundance was validated with Hyperion IMC, which also provided insight into the spatial organisation of VS. Both sporadic and *NF2*-SWN VS displayed regions of abnormal vasculature, recognized by SMA expression, as well as broad S100B expression for Schwann cells (Fig. 4A). Tumour-associated macrophages indicated by Iba1 staining were found in cases of both diffuse tissue infiltration and vascular-associated niches at high levels in *NF2*-SWN and sporadic VS, identifying tumour microenvironment heterogeneity within each VS sample.

Cell populations were segmented and clustered based on marker expression patterns of T-cell, macrophage, Schwann cell and vascular markers, with unidentifiable populations labelled as ‘Other’ (Fig. 4B). The T-cells present within both *NF2*-SWN and sporadic VS strongly expressed CD8a and CD3, macrophages expressed Iba1 and CD68, Schwann cells expressed S100B and vasculature expressed SMA. Dimensionality reduction of the marker expression assessed in Fig. 4B displayed close clustering of single cells within each group (Fig. 4C). Furthermore, the quantification of the absolute abundance of single cells and relative abundance of the cell type populations determined that macrophages and Schwann cells define the bulk of cells in the regions of interest of *NF2*-SWN and sporadic VS samples (Fig. 4D and E). Importantly, there were no significant differences in the absolute or relative abundances of the cell populations between *NF2*-SWN and sporadic VS. This observation was true across *NF2*-SWN and sporadic VS samples, with both comprising an average of 49% Schwann cells, as well as 31% and 36% macrophages, respectively (Fig. 4F).

Computational spatial mapping of the annotated single cells within the region of interest aligned well with the marker staining in Fig. 4A, thus validating the single-cell segmentation into distinct cell types (Fig. 4G). These data reinforced that the prominent macrophage infiltration resulted in dense macrophage-rich areas in both *NF2*-SWN and sporadic VS. Furthermore, the presence of T-cells in immune-rich niches was observed in both *NF2*-SWN and sporadic VS, indicating the similarities in their tumour immune microenvironments.

Together, the high level of S100B and Iba1 staining, as well as the subsequent quantification of cell type abundances identified using Hyperion IMC, confirms the strong Schwann cell and macrophage signatures identified using bulk transcriptomics in *NF2*-SWN and sporadic VS, as well as the similarities in tumour microenvironment components between these tumours.

## Discussion

The tumour microenvironment of VS contains heterogeneous regions of proliferative Schwann cells, abnormal vasculature, and tumour-infiltrating immune cells that promote immune-suppressive niches in regions of increased VS growth.^10^ Currently, immune-targeted therapeutics for VS treatment remain elusive with no clinical trials yet underway. However, promising drug targets are emerging such as COX-2,^10,48^ IL-1β, NLRP3^43,49^ and CSF1R^50,51^ for tumour-associated macrophages and the PD-1/PDL1 axis for T-cells.^20^ By assessing and contrasting key components of the tumour microenvironment of sporadic and *NF2*-SWN VS, this study aimed to compare the broad immune landscape of these tumours. As such, this study provides insight into the possibility of common treatments between patients with either *NF2*-SWN or sporadic VS, such that co-recruitment of both patient groups to clinical trials would be a reasonable approach. Likewise, evidence from one group of patients may be extrapolated to the other. Using bulk transcriptomic analyses and cellular deconvolution, followed by confirmation with the first use of high-dimensional Hyperion IMC in VS, this study identified a robust similarity between the tumour immune microenvironments of *NF2*-SWN and sporadic VS. Given the relative rarity of *NF2*-SWN compared with sporadic VS, this similarity could be used to carry out studies on the higher numbers of sporadic VS and, if potential efficacy is demonstrated, apply new and much needed treatment options in *NF2*-SWN. It is noteworthy that bevacizumab is now a standard treatment for schwannomas in *NF2*-SWN despite never being the subject of clinical trial.^52^ This was based on the urgent clinical need for new treatment. Clinical efficacy was subsequently demonstrated. This strong clinical need remains in *NF2*-SWN, and therefore, there is great potential for the rapid translation of a potential new drug to the clinical scenario, as occurred with bevacizumab. The similarity of tumour immune microenvironments allows the potential for any new drugs shown to be effective in *NF2*-SWN VS to be rolled out to sporadic VS.

When comparing *NF2*-SWN and sporadic VS to control nerve, this study determined S100 family and multiple fibrosis–related signalling pathways indicating the involvement of Schwann cells and fibroblasts in tumorigenesis. Close cell–cell interaction between Schwann cells and fibroblasts has been noted in VS and various cancers, with fibroblasts known to promote tumour growth by releasing growth factors and extensively secreting extracellular matrix components thus increasing the fibrotic nature of the VS tumour microenvironment.^32,53,54^ Importantly, this study identified no difference in the relative estimated abundance of fibroblasts between *NF2*-SWN and sporadic VS predicted by bulk RNA deconvolution, nor in the abundance of Schwann cells between these tumours by deconvolution and IMC.

In this study, both *NF2*-SWN and sporadic VS had upregulated immune-related gene expression, as well as pathways involving leukocyte recruitment and activation, which, in sporadic VS, has previously been linked to longer durations of clinical symptoms and tumour growth.^15,55^ The similarity in these signatures between *NF2*-SWN and sporadic VS alludes to an equivalent aetiology in these VS tumours driven by neuroinflammatory signalling. Leukocytes predicted to be present in the VS tumour immune microenvironment included mast cells and neutrophils, which were predicted to be few in number but have been found to correlate with tumour growth in cancer.^56,57^ For VS, a high neutrophil-to-lymphocyte ratio (NLR) in the blood of patients with VS is predictive of VS growth,^58^ indicating that the relative abundance of immune components within VS tumours may be more predictive of growth than the absolute abundance of certain populations.

Additionally, the present study identified T-cells within the VS tumour microenvironment, with no difference in relative abundance between *NF2*-SWN and sporadic samples. However, in a previous semi-quantitative immunohistochemical study by Tamura *et al.*,^14^ a difference in specific subtypes of T-cells, such as CD8+ cytotoxic T-cells, has been established between *NF2*-SWN and sporadic VS. Although no differences were enriched in T-cell–related pathways or in the abundance of T-cells in VS tissue in the present study, this highlights a need for a more complete T-cell characterization study in VS. This would be beneficial as T-cell–mediated responses may augment inflammation within the tumour microenvironment, where T-cell cytotoxicity has been shown to mediate pyroptotic cell death *in vitro* and *in vivo* in solid tumour mouse models, further inducing leukocyte recruitment.^59^ Hence, characterizing the roles of T-cells within the tumour microenvironment of VS tumours may yield anti-inflammatory therapeutic avenues for inhibition or augmentation.

The most notable immune signature in the VS samples from published Affymetrix datasets was linked to macrophages, with similar signalling pathways and gene expression when compared against nerve. However, the immune microenvironment of *NF2*-SWN compared to sporadic VS has previously only been assessed using histological and comparative low-dimensional imaging studies, which linked an increased density of macrophages to VS growth using immunohistochemical staining.^8,13^ This present study takes an important step forward with orthogonal analyses, revealing consistently prominent macrophage populations in VS through both transcriptomics and the first use of high-dimensional IMC in VS. Here, macrophages were found to constitute one-third of the total cells within VS tumours from both *NF2*-SWN and sporadic patients. The importance of this highly abundant population was emphasized in a previous study by Lewis *et al.*,^13^ where macrophages were specifically identified to account for 50–70% of the proliferating cells within VS. This was reinforced in the Xu *et al.*^32^ scRNA-seq data, where the only cell cluster to express proliferating markers TOP2A and MKI67 was macrophages.^32^ These studies indicate a highly active role of macrophages within the tumour microenvironment of VS.

Furthermore, Lewis *et al.*^13^ determined that the high density of macrophage cells within the tumour microenvironment correlated with regions of vasculature and VS growth. In the present investigation, there were no significant differences in macrophage abundances in *NF2*-SWN and sporadic VS determined by transcriptomics and imaging. Furthermore, the inflammatory profiles of these macrophages appear consistent between *NF2*-SWN and sporadic tumours when validated by qPCR in an additional cohort of VS samples. These inflammatory profiles include markers such as IL-1β, NLRP3, IL-6 and vascular endothelial growth factor, which have been implicated in VS progression by driving inflammation and angiogenesis within VS in niches of high cell division and tumour growth.^13,43,49,60^ Here, vascular-related cell abundances and gene expression were found to be comparable between *NF2*-SWN and sporadic VS tumours, and upon imaging, VS tissue showed similar niches of perivascular and diffuse tumour-associated macrophages. As such, the anti-vascular endothelial growth factor monoclonal antibody bevacizumab, used for the treatment of VS, may have dual functionality by reducing angiogenesis in *NF2*-SWN patients and therefore supressing tumour-associated macrophage infiltration, modifying the immunosuppressive microenvironment and reducing tumour growth in the perivascular regions determined in this study.^13^

In addition to VS tumour growth, macrophages have also been linked to sensorineural hearing loss (SNHL) in people with VS, which severely negatively impacts the quality of life experienced by these patients.^61,62^ Several studies demonstrate that the extent of SNHL in VS patients does not correlate with tumour volume, instead correlating with macrophage infiltration and inflammation during SNHL progression.^10,61,63^ Additionally, SNHL is increased in the contralateral ears of those with unilateral VS compared to the general population indicating the importance of systemic tumour-secreted inflammatory agents.^64^ Proinflammatory markers such as NLRP3, IL-18 and IL-1β have been identified in the progression of SNHL, and in this study, both *NF2*-SWN and sporadic VS expressed these markers at similar levels, as identified across the three published transcriptomic datasets and by qPCR.^43,49^ As such, immune-targeted therapeutics that reduce inflammatory signalling may concomitantly reduce the levels of SNHL experienced by both sporadic and *NF2*-SWN patients.

Overall, immune cells in the tumour immune microenvironment of VS have been implicated in the pathogenesis and significant symptomatic burden experienced by both sporadic and *NF2*-SWN VS patients. While importance lies in the individual contributions of immune cells to VS tumorigenesis, future studies into the interaction of these components within the tumour immune microenvironment of VS are likely to provide a more complete insight into the mechanisms underlying VS pathogenesis. Thus, the immune compartment of VS may provide strong candidates for therapeutic targeting, from whole cell populations, such as highly abundant macrophages, to specific cell–cell interactions, inflammatory pathways and cytokines.

## Conclusions

To conclude, the data generated in this study identify strong similarities in *NF2*-SWN and sporadic VS from their bulk transcriptomic profiles, enriched immune-related signalling pathways and overall abundances of tumour immune microenvironment components. This provides evidence for the use of sporadic samples to investigate *NF2*-SWN VS and a foundation for the hypothesis that immunotherapeutics may be equally effective in both sporadic and *NF2*-SWN VS tumours. The high abundance of tumour-associated macrophages and presence of T-cells noted in this study promote these immune cells as potential immunotherapeutic avenues to modify the tumour immune microenvironment in order to reduce tumour growth and SNHL in patients with either *NF2*-SWN or sporadic VS.

## Supplementary Material

fcad197_Supplementary_Data

## Data Availability

This study generated novel VS-specific signature matrix and Gene Expression Profile (GEP) files that have been made publicly available at the FigShare repository: Grace E Gregory, ‘CIBERSORTx Signature Matrix and GEP Files for Bulk RNA Transcriptomic Deconvolution to Predict Tumour Microenvironment Component Abundance’, University of Manchester, Workflow, https://doi.org/10.48420/21995978.v1.

## References

[1] Evans DGR. Neurofibromatosis type 2 (NF2): A clinical and molecular review. Orphanet J Rare Dis. 2009;4(1). 10.1186/1750-1172-4-16 PMC2708144 19545378

[2] Lassaletta L, Torres-Martín M, Peña-Granero C, et al NF2 Genetic alterations in sporadic vestibular schwannomas: Clinical implications. Otol Neurotol. 2013;34(7):1355–1361. 23921927 10.1097/MAO.0b013e318298ac79

[3] Agnihotri S, Jalali S, Wilson MR, et al The genomic landscape of schwannoma. Nat Genet. 2016;48(11):1339–1348. 27723760 10.1038/ng.3688

[4] Hadfield KD, Smith MJ, Urquhart JE, et al Rates of loss of heterozygosity and mitotic recombination in NF2 schwannomas, sporadic vestibular schwannomas and schwannomatosis schwannomas. Oncogene. 2010;29(47):6216–6221. 20729918 10.1038/onc.2010.363

[5] Sadler KV, Bowers NL, Hartley C, et al Sporadic vestibular schwannoma: A molecular testing summary. J Med Genet. 2021;58(4):227–233. 32576656 10.1136/jmedgenet-2020-107022

[6] Ren Y, Chari DA, Vasilijic S, Welling DB, Stankovic KM. New developments in neurofibromatosis type 2 and vestibular schwannoma. Neuro-Oncology Adv. 2021;3(1). 10.1093/noajnl/vdaa153 PMC7881257 33604573

[7] Gregory GE, Islim AI, Hannan CJ, et al The clinical, genetic, and immune landscape of meningioma in patients with NF2-schwannomatosis. Neuro-Oncology Adv. 2023;5(Supplement_1):i94–i104. 10.1093/noajnl/vdac127 PMC10243851 37287576

[8] Sobel RA, Wang Y. Vestibular (acoustic) schwannomas: Histologic features in neurofibromatosis 2 and in unilateral cases. J Neuropathol Exp Neurol. 1993;52(2):106–113. 8440992 10.1097/00005072-199303000-00002

[9] Torres-Martin M, Lassaletta L, Isla A, et al Global expression profile in low grade meningiomas and schwannomas shows upregulation of PDGFD, CDH1 and SLIT2 compared to their healthy tissue. Oncol Rep. 2014;32(6):2327–2334. 25333347 10.3892/or.2014.3526 PMC4240498

[10] Hannan CJ, Lewis D, O’Leary C, et al The inflammatory microenvironment in vestibular schwannoma. Neuro-Oncology Adv. 2020;2(1):vdaa023. 10.1093/noajnl/vdaa023 PMC7212860 32642684

[11] Pathmanaban ON, Sadler KV, Kamaly-Asl ID, et al Association of genetic predisposition with solitary schwannoma or meningioma in children and young adults. JAMA Neurol. 2017;74(9):1123–1129. 28759666 10.1001/jamaneurol.2017.1406 PMC5710179

[12] Evans DG, Hartley CL, Smith PT, et al Incidence of mosaicism in 1055 de novo NF2 cases: Much higher than previous estimates with high utility of next-generation sequencing. Genet Med. 2020;22(1):53–59. 31273341 10.1038/s41436-019-0598-7

[13] Lewis D, Donofrio CA, O’Leary C, et al The microenvironment in sporadic and neurofibromatosis type II–related vestibular schwannoma: The same tumor or different? A comparative imaging and neuropathology study. J Neurosurg. 2021;134(5):1419–1429. 10.3171/2020.3.JNS193230 32470937

[14] Tamura R, Morimoto Y, Sato M, et al Difference in the hypoxic immunosuppressive microenvironment of patients with neurofibromatosis type 2 schwannomas and sporadic schwannomas. J Neurooncol. 2020;146(2):265–273. 31897926 10.1007/s11060-019-03388-5

[15] Labit-Bouvier C, Crebassa B, Bouvier C, Andrac-Meyer L, Magnan J, Charpin C. Clinicopathologic growth factors in vestibular schwannomas: A morphological and immunohistochemical study of 69 tumours. Acta Otolaryngol. 2000;120(8):950–954. 11200590 10.1080/00016480050218681

[16] Lewis D, Roncaroli F, Agushi E, et al Inflammation and vascular permeability correlate with growth in sporadic vestibular schwannoma. Neuro Oncol. 2019;21(3):314–325. 30388263 10.1093/neuonc/noy177 PMC6380424

[17] de Vries M, Briaire-de Bruijn I, Malessy MJA, de Bruïne SFT, van der Mey AGL, Hogendoorn PCW. Tumor-associated macrophages are related to volumetric growth of vestibular schwannomas. Otol Neurotol. 2013;34(2):347–352. 23295727 10.1097/MAO.0b013e31827c9fbf

[18] Hannan CJ, Lewis D, O’Leary C, et al Increased circulating chemokines and macrophage recruitment in growing vestibular schwannomas. Neurosurgery. 2022;92(3):581–589 Ahead of Print. 36729787 10.1227/neu.0000000000002252

[19] Rossi ML, Jones NR, Esiri MM, Havas L, Nakamura N, Coakham HB. Mononuclear cell infiltrate, HLA-Dr expression and proliferation in 37 acoustic schwannomas. Histol Histopathol. 1990;5(4):427–432. 2134396

[20] Wang S, Liechty B, Patel S, et al Programmed death ligand 1 expression and tumor infiltrating lymphocytes in neurofibromatosis type 1 and 2 associated tumors. J Neurooncol. 2018;138(1):183–190. 29427150 10.1007/s11060-018-2788-6 PMC5930071

[21] Tamura R, Toda M. A critical overview of targeted therapies for vestibular schwannoma. Int J Mol Sci. 2022;23(10):5462. 35628268 10.3390/ijms23105462 PMC9143502

[22] Hannan CJ, Lewis D, O’Leary C, et al Beyond antoni: A surgeon’s guide to the vestibular schwannoma microenvironment. J Neurol Surg B Skull Base. 2020;83(1):1–10. 35155063 10.1055/s-0040-1716688 PMC8824628

[23] Steen CB, Liu CL, Alizadeh AA, Newman AM. Profiling cell type abundance and expression in bulk tissues with CIBERSORTx. Methods Mol Biol. 2020;2117:135–157. 31960376 10.1007/978-1-0716-0301-7_7 PMC7695353

[24] Gregory GE. CIBERSORTx signature matrix and GEP files for bulk RNA transcriptomic deconvolution to predict tumour microenvironment component abundance. Univ Manchester. 2023;V1.

[25] Zhao Y, Liu P, Zhang N, et al Targeting the cMET pathway augments radiation response without adverse effect on hearing in NF2 schwannoma models. Proc Natl Acad Sci. 2018;115(9):E2077–E2084. 29440379 10.1073/pnas.1719966115 PMC5834719

[26] Gugel I, Ebner FH, Grimm F, et al Contribution of mTOR and PTEN to radioresistance in sporadic and NF2-associated vestibular schwannomas: A microarray and pathway analysis. Cancers (Basel). 2020;12(1):177. 31936793 10.3390/cancers12010177 PMC7016954

[27] MacDonald JW. Affycoretools: Functions useful for those doing repetitive analyses with Affymetrix GeneChips (R package version 1.68.1.). Bioconductor. Published online 2022. Accessed June 16, 2022. https://www.bioconductor.org/packages/release/bioc/html/affycoretools.html

[28] Carvalho B. pd.hugene.1.0.st.v1: Platform Design Info for Affymetrix HuGene-1_0-st-v1. R package version 3.14.1. Bioconductor. Published online 2015. Accessed June 16, 2022. https://bioconductor.org/packages/release/data/annotation/html/pd.hugene.1.0.st.v1.html

[29] MacDonald JW. pd.hta.2.0: Platform Design Info for Affymetrix HTA-2_0. R package version 3.12.2. *Bioconductor*. Published online 2017. Accessed June 16, 2022. https://bioconductor.org/packages/release/data/annotation/html/pd.hta.2.0.html

[30] Carvalho B. pd.hg.u219: Platform Design Info for The Manufacturer’s Name HG-U219. R package version 3.12.0. Bioconductor. Published online 2015. Accessed June 16, 2022. http://bioconductor.org/packages/release/data/annotation/html/pd.hg.u219.html

[31] Ritchie ME, Phipson B, Wu D, et al Limma powers differential expression analyses for RNA-sequencing and microarray studies. Nucleic Acids Res. 2015;43(7):e47. 25605792 10.1093/nar/gkv007 PMC4402510

[32] Xu M, Wang S, Jiang Y, et al Single-cell RNA-Seq reveals heterogeneity of cell communications between Schwann cells and fibroblasts within vestibular schwannoma microenvironment. Am J Pathol. 2022;192(9):1230–1249. 35750260 10.1016/j.ajpath.2022.06.006

[33] Newman AM, Liu CL, Green MR, et al Robust enumeration of cell subsets from tissue expression profiles. Nat Methods. 2015;12(5):453–457. 25822800 10.1038/nmeth.3337 PMC4739640

[34] Chen B, Khodadoust MS, Liu CL, Newman AM, Alizadeh AA. Profiling tumor infiltrating immune cells with CIBERSORT. Methods Mol Biol. 2018;1711:243. 29344893 10.1007/978-1-4939-7493-1_12 PMC5895181

[35] Zanotelli VR, Bodenmiller B. Imcsegmentationpipeline: A pixel-classification based multiplexed image segmentation pipeline. Zenodo; 2017. doi:10.5281/zenodo.3841961

[36] Berg S, Kutra D, Kroeger T, et al Ilastik: Interactive machine learning for (bio)image analysis. Nat Methods. 2019;16(12):1226–1232. 31570887 10.1038/s41592-019-0582-9

[37] Wolf FA, Angerer P, Theis FJ. SCANPY: Large-scale single-cell gene expression data analysis. Genome Biol. 2018;19(1):1–5. 29409532 10.1186/s13059-017-1382-0 PMC5802054

[38] Benjamini Y, Hochberg Y. Controlling the false discovery rate: A practical and powerful approach to multiple testing. J R Stat Soc Ser B. 1995;57(1):289–300.

[39] Fujiwara S, Hoshikawa S, Ueno T, et al SOX10 Transactivates S100B to suppress Schwann cell proliferation and to promote myelination. PLoS One. 2014;9(12):e115400. 10.1371/journal.pone.0115400 PMC4275212 25536222

[40] Triolo D, Dina G, Taveggia C, et al Vimentin regulates peripheral nerve myelination. Development. 2012;139(7):1359–1367. 22357929 10.1242/dev.072371

[41] Veith AP, Henderson K, Spencer A, Sligar AD, Baker AB. Therapeutic strategies for enhancing angiogenesis in wound healing. Adv Drug Deliv Rev. 2019;146:97–125. 30267742 10.1016/j.addr.2018.09.010 PMC6435442

[42] Schmitt HA, Pich A, Prenzler NK, et al Personalized proteomics for precision diagnostics in hearing loss: Disease-specific analysis of human perilymph by mass spectrometry. ACS Omega. 2021;6(33):21241–21254. 34471729 10.1021/acsomega.1c01136 PMC8387986

[43] Sagers JE, Sahin MI, Moon I, et al NLRP3 inflammasome activation in human vestibular schwannoma: Implications for tumor-induced hearing loss. Hear Res. 2019;381:107770. 10.1016/j.heares.2019.07.007 31430634

[44] Mujal AM, Combes AJ, Rao AA, et al Holistic characterization of tumor monocyte-to-macrophage differentiation integrates distinct immune phenotypes in kidney cancer. Cancer Immunol Res. 2022;10(4):403–419. 35181780 10.1158/2326-6066.CIR-21-0588 PMC8982148

[45] Deichaite I, Sears TJ, Sutton L, et al Differential regulation of TNFα and IL-6 expression contributes to immune evasion in prostate cancer. J Transl Med. 2022;20(1):527. 36371231 10.1186/s12967-022-03731-x PMC9652804

[46] Modak M, Mattes AK, Reiss D, et al CD206 + tumor-associated macrophages cross-present tumor antigen and drive antitumor immunity. JCI Insight. 2022;7(11):e155022. 10.1172/jci.insight.155022 PMC9220841 35503656

[47] Gieryng A, Pszczolkowska D, Walentynowicz KA, Rajan WD, Kaminska B. Immune microenvironment of gliomas. Lab Investig. 2017;97(5):498–518. 28287634 10.1038/labinvest.2017.19

[48] Behling F, Ries V, Skardelly M, et al COX2 expression is associated with proliferation and tumor extension in vestibular schwannoma but is not influenced by acetylsalicylic acid intake. Acta Neuropathol Commun. 2019;7(1):105. 31291992 10.1186/s40478-019-0760-0 PMC6621994

[49] Gregory GE, Munro KJ, Couper KN, Pathmanaban ON, Brough D. The NLRP3 inflammasome as a target for sensorineural hearing loss. Clin Immunol. 2023;249:109287. 10.1016/j.clim.2023.109287 36907540

[50] Ries CH, Cannarile MA, Hoves S, et al Targeting tumor-associated macrophages with anti-CSF-1R antibody reveals a strategy for cancer therapy. Cancer Cell. 2014;25(6):846–859. 24898549 10.1016/j.ccr.2014.05.016

[51] de Vries WM, Briaire-de Bruijn IH, van Benthem PPG, van der Mey AGL, Hogendoorn PCW. M-CSF and IL-34 expression as indicators for growth in sporadic vestibular schwannoma. Virchows Arch. 2019;474(3):375–381. 30580386 10.1007/s00428-018-2503-1 PMC6515692

[52] Plotkin SR, Stemmer-Rachamimov AO, Barker FG, et al Hearing improvement after bevacizumab in patients with neurofibromatosis type 2. N Engl J Med. 2009;361(4):358–367. 19587327 10.1056/NEJMoa0902579 PMC4816642

[53] Ermakov MS, Nushtaeva AA, Richter VA, Koval OA. Cancer-associated fibroblasts and their role in tumor progression. Vavilov J Genet Breed. 2022;26(1):14–21. 10.18699/VJGB-22-03 PMC8894099 35342854

[54] Olbrecht S, Busschaert P, Qian J, et al High-grade serous tubo-ovarian cancer refined with single-cell RNA sequencing: Specific cell subtypes influence survival and determine molecular subtype classification. Genome Med. 2021;13(1):1–30. 34238352 10.1186/s13073-021-00922-x PMC8268616

[55] de Vries M, Hogendoorn PCW, Briaire-de Bruyn I, Malessy MJA, van der Mey AGL. Intratumoral hemorrhage, vessel density, and the inflammatory reaction contribute to volume increase of sporadic vestibular schwannomas. Virchows Arch. 2012;460(6):629–636. 22555941 10.1007/s00428-012-1236-9 PMC3371334

[56] Lv Y, Zhao Y, Wang X, et al Increased intratumoral mast cells foster immune suppression and gastric cancer progression through TNF-α-PD-L1 pathway. J Immunother Cancer. 2019;7(1):54. 30808413 10.1186/s40425-019-0530-3 PMC6390584

[57] Jaillon S, Ponzetta A, Di Mitri D, Santoni A, Bonecchi R, Mantovani A. Neutrophil diversity and plasticity in tumour progression and therapy. Nat Rev Cancer. 2020;20(9):485–503. 32694624 10.1038/s41568-020-0281-y

[58] Kontorinis G, Crowther JA, Iliodromiti S, Taylor WAS, Locke R. Neutrophil to lymphocyte ratio as a predictive marker of vestibular schwannoma growth. Otol Neurotol. 2016;37(5):580–585. 27093024 10.1097/MAO.0000000000001026

[59] Zhou Z, He H, Wang K, et al Granzyme A from cytotoxic lymphocytes cleaves GSDMB to trigger pyroptosis in target cells. Science. 2020;368(6494):eaaz7548. 10.1126/science.aaz7548 32299851

[60] Taurone S, Bianchi E, Attanasio G, et al Immunohistochemical profile of cytokines and growth factors expressed in vestibular schwannoma and in normal vestibular nerve tissue. Mol Med Rep. 2015;12(1):737–745. 25738867 10.3892/mmr.2015.3415

[61] Nisenbaum E, Misztal C, Szczupak M, et al Tumor-associated macrophages in vestibular schwannoma and relationship to hearing. OTO Open. 2021;5(4):2473974X2110591. 10.1177/2473974X211059111 PMC8638079 34870062

[62] Neary WJ, Hillier VF, Flute T, Stephens D, Ramsden RT, Evans DGR. Use of a closed set questionnaire to measure primary and secondary effects of neurofibromatosis type 2. J Laryngol Otol. 2010;124(7):720–728. 20219149 10.1017/S0022215110000460

[63] Brown A, Early S, Vasilijic S, Stankovic KM. Sporadic vestibular schwannoma size and location do not correlate with the severity of hearing loss at initial presentation. Front Oncol. 2022;12:836504. 10.3389/fonc.2022.836504 PMC8965062 35372070

[64] Early S, Rinnooy Kan CE, Eggink M, Frijns JHM, Stankovic KM. Progression of contralateral hearing loss in patients with sporadic vestibular schwannoma. Front Neurol. 2020;11:796. 33013614 10.3389/fneur.2020.00796 PMC7461819

