# Supplementary material for: The comparable tumour microenvironment in sporadic and NF2-related schwannomatosis vestibular schwannoma
Source: Brain Commun. 2023 Jul 21;5(4):fcad197. doi: 10.1093/braincomms/fcad197 (PMC10481781; doi:10.1093/braincomms/fcad197)
Supplement: fcad197_Supplementary_Data [file fcad197_Supplementary_Data.pdf]

SUPPLEMENTARY INFORMATION

Compartment	Acronym	Name
Schwann Cell	S100B	S100 calcium-binding protein B
	GPM6B	Glycoprotein M6B
	GFRA3	GDNF family receptor alpha-3
	CCND1	Cyclin D1
	GPX3	Glutathione peroxidase 3
	VEGFA	Vascular endothelial growth factor A
	NCMAP	Non-compact myelin associated protein
	PRX	Periaxin
Macrophage	C1QC	Complement C1q C chain
	GPR34	G protein-coupled receptor 34
	SPP1	Secreted phosphoprotein 1
	HSPA1A/B	Heat shock 70 kDa protein 1
	SLC2A5	Solute carrier family 2 member 5
	CX3CR1	C-X3-C motif chemokine receptor 1
	PDK4	Pyruvate dehydrogenase kinase 4
	GPNMB	Glycoprotein NMB
	MTRNR2L12	MT-RNR2 Like 12
	TOP2A	DNA topoisomerase II-alpha
	MKI67	Marker of proliferation Ki-67
	MT-CO1	Mitochondrially encoded cytochrome C oxidase 1
	MT-CO2	Mitochondrially encoded cytochrome C oxidase 2
	MT-CO3	Mitochondrially encoded cytochrome C oxidase 3
Vascular smooth muscle cells	ACTA2	Actin alpha 2
	TAGLN	Transgelin
Fibroblasts	DCN	Decorin
	CYP1B1	Cytochrome P450 family 1 subfamily B member 1
Endothelial cells	PLVAP	Plasmalemma vesicle associated protein
	FLT1	Fms related receptor tyrosine kinase 1
T-cells	CCL5	C-C motif chemokine ligand 5
	TRBC2	T cell receptor beta constant 2
Mast cells	TPSAB1	Tryptase alpha/beta 1
	TPSB2	Tryptase beta 2
Neutrophils	S100A9	S100 calcium binding protein A9
	S100A8	S100 calcium binding protein A8

Supplementary Table 1 Genes used to manually annotate single cell RNA-seq clusters. Published single cell RNA sequencing data from three sporadic VS samples from Xu *et al*. (2022)[33] were obtained from the National Omics Data Encyclopaedia (https://www.biosino.org/node; accession code OEP001871)

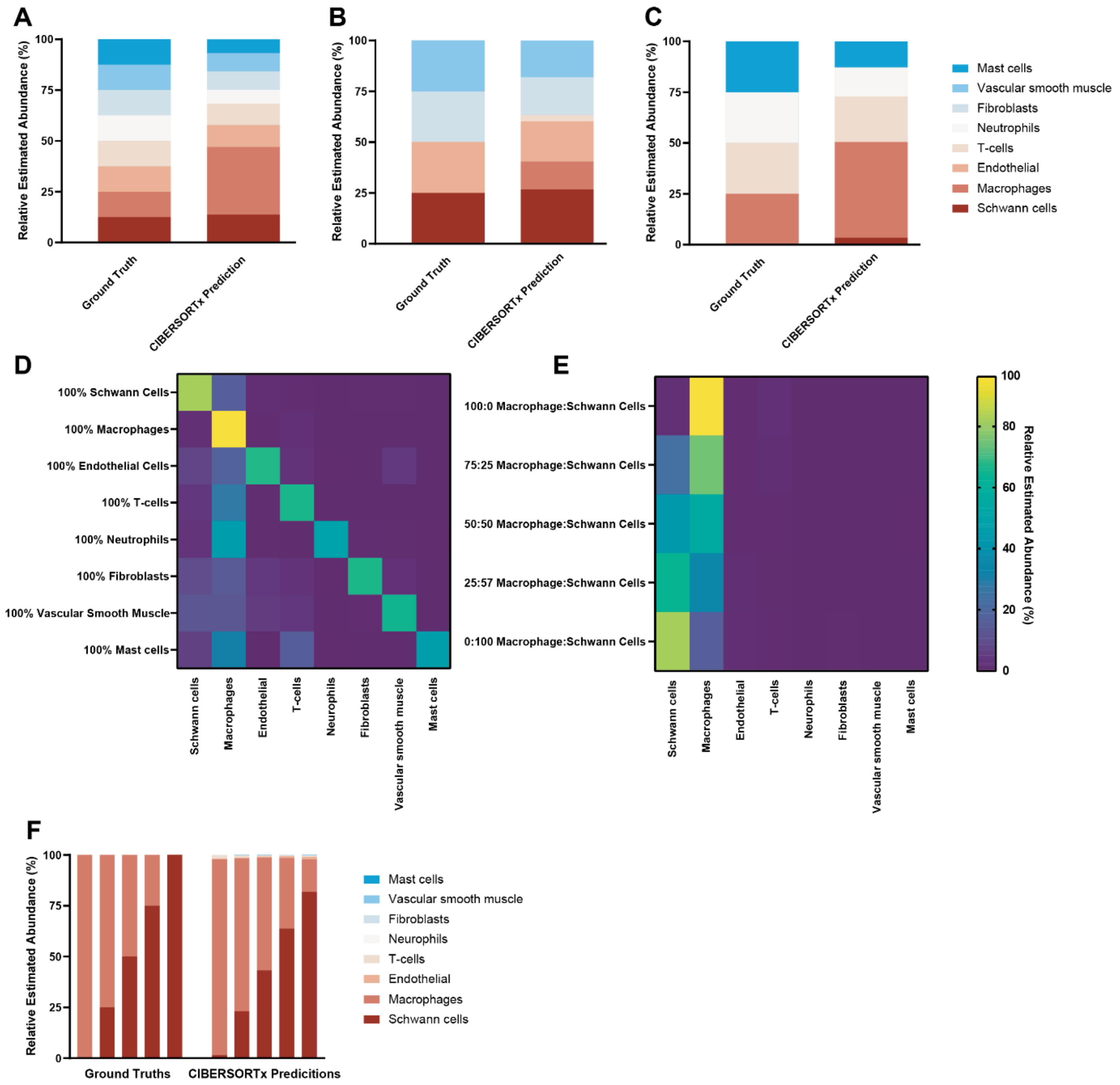

Supplementary Fig.1 The CIBERSORTx signature matrix specific to vestibular schwannoma (VS) tumours can be used to accurately deconvolve bulk transcriptomic data. Published single cell RNA sequencing data from three sporadic VS samples from Xu *et al*. (2022)[33] were obtained from the National Omics Data Encyclopaedia (https://www.biosino.org/node; accession code OEP001871). **(A-C)** CIBERSORTx prediction of relative estimated abundance of cell-type populations was used to identify cell populations present within the ground truth pseudo-bulk data. **(D)** Relative estimated abundance of single-population pseudo-bulk data. **(E-F)** The relative estimated abundances of pseudo-bulk data from different proportions of macrophages:Schwann cells.

Compartment	Acronym	Antigen	Target	Clone	Supplier
Schwann Cell	S100B	S100 calcium-binding protein B	Schwann cell	EP1576Y	Abcam (ab215989)
	Pan-Cytokeratin	-	Tumour cells	AE-1/AE-3	BioLegend (914204)
	Vimentin	-	Tumour cells	RV202	Novus Biologicals (NBP1-97672)
Immune	Iba1	Ionised calcium-binding adaptor molecule 1	Macrophages	Polyclonal	FUJIFILM Wako Pure Chemical Corp (019-19741)
	CD68	-	Macrophages	KP1	BioLegend (916104)
	CX3CR1	Fractalkine receptor	Immune cells	Polyclonal	Abcam (ab8020)
	CD8a	CD8 alpha chain	CD8 T-cells	C8/144B	eBioscience (14-0085-82)
	CD3	-	T-cells	D7A6E (sporadic), CD3-12 (NF2)	Cell Signalling Technology (85061), Bio-Rad (MCA1477)
Vasculature	SMA	Smooth muscle actin	Smooth muscle cells	1A4	Bio-Rad (MCA5781GA)
	CD31	Platelet endothelial cell adhesion molecule 1	Endothelial cells	JC/70A	Novus Biologicals (NB600-562)
	vWF	Von Willebrand factor	Endothelial cells	Polyclonal	DAKO, Agilent (A0082)

Supplementary Table 2 Antibody panel for Hyperion imaging mass cytometry.

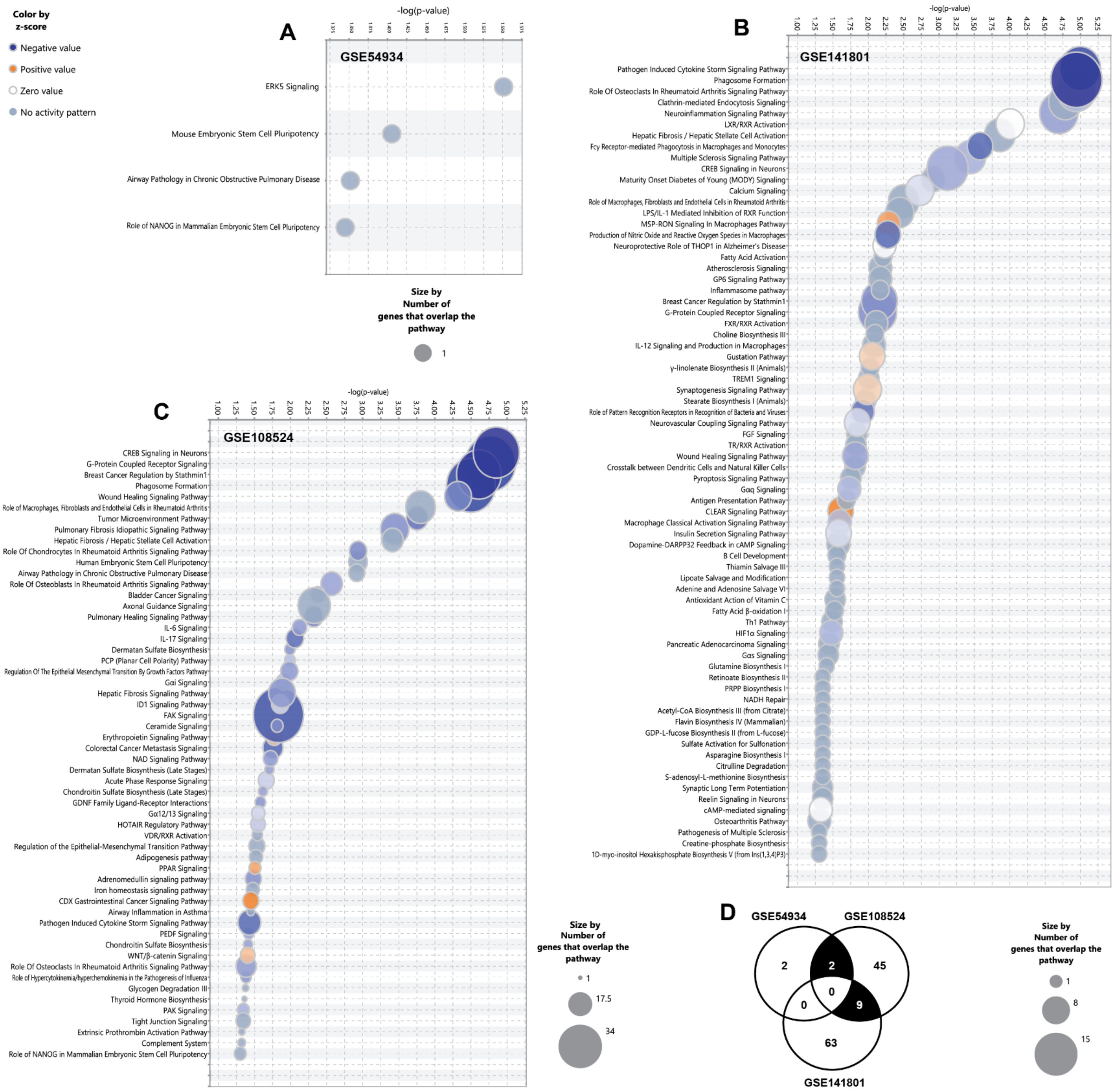

Supplementary Fig.2 Differentially expressed genes (DEG) between *NF2*-schwannomatosis and sporadic vestibular schwannoma (VS) are unlikely to represent a conserved, biologically relevant difference in the tumours. GSE54934, GSE108524 and GSE141801 available from Gene Expression Omnibus. **(A-C)** Significantly enriched signalling pathways using Ingenuity Pathway Analysis (IPA) from *NF2*-SWN compared to sporadic VS. **(D)** Overlap of the significantly enriched signalling pathways from IPA observed between the three datasets. P-value ≤ 0.05.
